# Carcinome papillaire de la thyroïde sur maladie de Basedow

**DOI:** 10.11604/pamj.2015.21.238.6707

**Published:** 2015-07-31

**Authors:** Madiha Mahfoudhi, Khaled Khamassi

**Affiliations:** 1 Service de Médecine Interne A, Hôpital Charles Nicolle, Tunis, Tunisie; 2 Service ORL, Hôpital Charles Nicolle, Tunis, Tunisie

**Keywords:** Carcinome papillaire, thyroïde, maladie de Basedow, papillary carcinoma, thyroid, Basedow disease

## Image en medicine

La survenue d'une maladie de Basedow n’élimine pas la possibilité d'un cancer thyroïdien associé. Le carcinome thyroïdien sur maladie de Basedow est particulièrement rare (1 à 2%). Le plus souvent, il s'agit d'un carcinome papillaire. Celui-ci est le plus souvent de découverte fortuite. Patient âgé de 31 ans sans antécédents pathologiques notables a été hospitalisé pour une tuméfaction basi-cervicale antérieure, associée à des signes cliniques d'hyperthyroïdie, sans signes de compression. L'examen physique a objectivé une exophtalmie bilatérale et un goitre stade III. La thyroïde était augmentée de taille sans nodule évident, avec un souffle vasculaire en regard. Les aires ganglionnaires étaient libres. Le bilan hormonal a révélé un profil d'une hyperthyroïdie périphérique avec une TSH effondrée. L’échographie cervicale a trouvé une thyroïde augmentée de taille homogène dans son ensemble, hyper vascularisée et anodulaire. Le traitement s'est basé au départ sur l'administration d'antithyroïdiens de synthèse. L’évolution était marquée par la persistance de l'hyperthyroïdie clinique et biologique. Plusieurs pathologies peuvent s'associer exceptionnellement à une maladie de Basedow tel qu'un lymphome, une tuberculose ou un carcinome. Il a bénéficié d'une thyroïdectomie totale. L'aspect macroscopique de la thyroïde était évocateur d'une maladie de Basedow. L'examen anatomopathologique a révélé un carcinome papillaire de 13 mm du lobe droit associé à un parenchyme thyroïdien remanié compatible avec une maladie de Basedow. Une Irathérapie complémentaire (1 cure de 100 mCi) lui a été instaurée. L’évolution était favorable sans notion de récidive ou d'extension. Le recul était de 2 ans.

**Figure 1:**
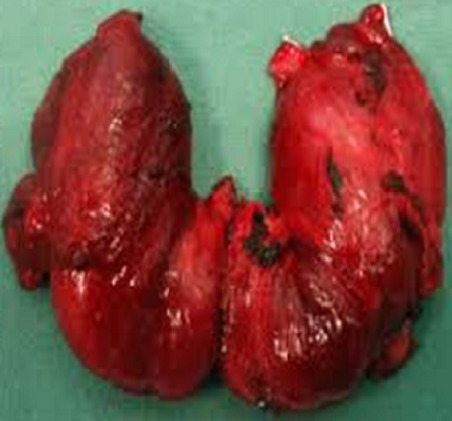
Pièce de thyroïdectomie montrant un aspect évocateur d'une maladie de Basedow

